# Clindamycin-Loaded Halloysite Nanotubes as the Antibacterial Component of Composite Hydrogel for Bone Repair

**DOI:** 10.3390/polym14235151

**Published:** 2022-11-26

**Authors:** Adrianna Machowska, Joanna Klara, Gabriela Ledwójcik, Kinga Wójcik, Joanna Dulińska-Litewka, Anna Karewicz

**Affiliations:** 1 Department of Chemistry, Jagiellonian University, 2 Gronostajowa St., 30-387 Kraków, Poland; 2 Department of Microbiology, Faculty of Biochemistry, Biophysics and Biotechnology, Jagiellonian University, Gronostajowa 7, 30-387 Kraków, Poland; 3 Chair of Medical Biochemistry, Faculty of Medicine, Medical College, Jagiellonian University, 31-034 Kraków, Poland

**Keywords:** halloysite, clindamycin, hydrogel crosslinking, alginate, collagen, β-TCP

## Abstract

A new drug delivery system consisting of clindamycin phosphate entrapped in acid-etched halloysite nanotubes was successfully prepared and characterized. It was then used as an antibacterial component of the multicomponent hydrogel designed as a material for bone regeneration. First, halloysite (HNT) was etched and clindamycin phosphate (CP) was entrapped in both raw and modified nanotubes, resulting in HNT-CP and EHNT-CP systems. They were characterized using SEM, TEM, TGA and FTIR; the entrapment efficiency and release of CP from both systems were also studied. EHNT-CP was then used as an antibacterial component of the two hydrogels composed of alginate, collagen and β-TCP. The hydrogels were prepared using different crosslinking procedures but had the same composition. The morphology, porosity, degradation rate, CP release profile, cytocompatibility, antibacterial activity and ability to induce biomineralization were studied for both materials. The hydrogel obtained by a chemical crosslinking with EDC followed by the physical crosslinking with calcium ions had better properties and was shown to have potential as a bone repair material.

## 1. Introduction

Artificial bone scaffolds are becoming a solution of choice in the treatment of bone defects. They no longer serve solely as a replacement material but are rather a temporary support allowing the bone tissue to regenerate. Various bioactive components are therefore introduced into the scaffolds. Their role is not only to promote the osteogenesis but also to prevent infection after the bone defect was exposed (due to the trauma or the surgery) to various gems. Among the more serious and difficult-to-prevent infections occurring as a result of the surgery are those caused by hospital bacteria, such as *Staphylococcus aureus*. Antibiotics used to prevent such infections are either administered systemically or locally as components of the scaffold. Most of the research conducted so far was focused solely on either designing the highly biocompatible materials promoting cell adhesion and osteogenesis or on the antibacterial materials dedicated to treat infections. Collagen, as a component of natural bone tissue [1] with a vital role in the biomineralization processes [2], and its derivative gelatin are the most logical choices to form osteoconductive materials. Some of the other polysaccharides with structural similarity to glycosaminoglycans (GAG), such as chitosan or hyaluronic acid, are also often proposed as components of scaffolds for bone regeneration. These polymers are frequently mixed with sodium alginate due to its broad availability and ability to form, in mild conditions, physical hydrogels. An interesting approach was proposed by Dubruel and coworkers [3], where copolymers of methacrylated gelatin and alginate were used to form bone implants that are cross-linkable via three different routes: mediated by temperature, mediated by UV irradiation (using a photoinitiator) and mediated by calcium ions. The biocompatibility of the resulting materials was improved by an increase in the gelatin content, while their storage modulus could be increased and precisely adjusted by adding various amounts of calcium ions. Collagen, chitosan and hyaluronic acid-based hydrogels cross-linked by genipin were used to obtain an injectable material for bone regeneration with promising properties [4]. A comprehensive review on these materials was given by Gomes and coworkers [5].

To reinforce the relatively weak hydrogels obtained from natural polymers, β-tricalcium phosphate (β-TCP) is often used [6,7], as it is known to provide not only improved mechanical properties but also osteoconductivity [8]. For example, the 50/50 alginate/β-TCP nanocomposite scaffolds obtained by rapid prototyping showed not only a well-defined morphology and resistance to compression comparable to that of trabecular bone but also excellent biological properties, facilitating bioadhesion [7]. Other inorganic materials commonly used to strengthen hydrogels for tissue engineering include bioglass [9], silica [10,11] and nanoclays, especially halloysite [12,13]. 

Halloysite is a nanoclay naturally occurring in a unique form of nanotubes which are ca. 0.2–1 μm in length and have an inner diameter in the range of 15–100 nm. Halloysite nanotubes (HNT) are biocompatible and exhibit low cytotoxicity [14]; therefore, they have found a broad range of biomedical applications, such as drug delivery and the reinforcement of polymeric biomaterials [15]. Recently, HNTs were proposed as a component of various hydrogel materials for bone repair. The studies on the composite scaffolds made from chitosan and its mixture with gelatin and agarose, reinforced with 3–6% of HNT, showed that HNTs have good compatibility with chitosan and improve not only the mechanical properties but also the wettability of the material [16]. The presence of HNT did not negatively influence the biocompatibility of the material and promoted cell adhesion. The nanocomposite of polycaprolactone-polyethylene glycol-polycaprolactone/gelatin and HNT (PCEC/Gel/HNT) promoted the differentiation of stem cells (hDPSCs) [17]. The presence of nanotubes increased the surface and its roughness, facilitating the scaffold’s interactions with cells. The studies of the nanocomposite scaffold obtained from methacrylated gelatin and HNT also indicated the supporting role of the nanotubes in hDPSCs differentiation through the upregulation of the osteogenic differentiation-related genes [18].

Various bone-filling materials containing antimicrobial components were also proposed in the literature, with the purpose being to prevent perioperative infections. Silver nanoparticles are one of the choices [19]; however, their long-term safety is still a matter of debate [20]. Chitosan [4] and its quaternized derivative [21] were also reported to exhibit antibacterial properties and thus may serve as a bioactive component of the scaffold. The studies on its activity are, however, still ongoing, and the polymer may only serve to protect the implant itself, not its environment. The most intensely studied systems are those based on antibiotics, released locally in a controlled way. The slow release allows for the constant support of the environment of the implant with an antimicrobial drug, while not exceeding the local concentration that could be toxic or significantly hinder bone regeneration. HNT was already reported as a carrier of antibiotics [22].

While the literature reports on materials either promoting bone regeneration or preventing perioperative infections are abundant, the materials with both characteristics are less frequently studied. There are few reports on using HNTs as a scaffold component to deliver antibiotics locally. Gentamycin-loaded HNTs were used in PCEC/Gel material [23], while vancomycin-loaded HNTs were used in silk fibroin scaffolds [24]. To the best of our knowledge, however, clindamycin-loaded HNTs were not used as a component of the materials for bone repair. Clindamycin is a semisynthetic, more potent derivative of lincomycin. It is widely used to treat osteoarticular diseases, especially those caused by *Staphylococcus aureus*, as it effectively penetrates the bone tissue and often prevents the adhesion of bacteria, as well as the biofilm formation [25]. Low doses of clindamycin were found to be sufficient in a successful decontamination of the autogenous particulate bone grafts [26]. The antibiotic was also shown to promote cell differentiation in low concentrations [27]. Clindamycin was also proposed in the treatment of osteomyelitis, an inflammatory bone disease caused by infection, which is often a result of long-bone fractures [28]. Due to the necessity of prolonged systemic therapy with high doses of the antibiotic, the possibility of the local delivery of clindamycin from the scaffold was recently studied [29]. This approach is further supported by the fact that osteomyelitis, especially in its chronic form, requires surgical removal of the necrotic bone tissue and its replacement. Our studies aim at the development of a hydrogel scaffold with clindamycin-loaded HNT as an antibacterial component, allowing for the controlled, local delivery of this antibiotic. The proposed material fits within a relatively less studied category of materials designed for both the prevention of perioperative infections and the promotion of bone regeneration. 

As HNT nanotubes have positively charged lumen, there is the possibility of using electrostatic interactions to selectively entrap the negatively charged drug inside them. We have, therefore, introduced clindamycin phosphate into the HNT and used an HNT-clindamycin phosphate system (HNT-CP) as a bioactive component of the bone scaffold. To increase the CP loading, we have etched the lumen of the nanotubes prior to the antibiotic’s entrapment. The obtained HNT-CP system was characterized, and its possible application as an antibacterial component of a multifunctional hydrogel composed of alginate, collagen and β-TCP, with possible applications in bone repair, was studied.

## 2. Materials and Methods

### 2.1. Materials

Halloysite (HNT) nanoclay, medium size: 30–70 nm × 1–3 μm (Sigma-Aldrich, Poznań, Poland); clindamycin phosphate (CP) Pharmaceutical Secondary Standard; Certified Reference Material, 95.2% (Sigma-Aldrich, Poznań, Poland); tricalcium β-phosphate, ≥98.8%, (Sigma-Aldrich, Poznań, Poland); sodium alginate, medium viscosity (Sigma-Aldrich, Poznań, Poland); type I collagen from rat tail, Corning (VWR International, Poland); N-hydroxysuccinimide (NHS), 98% (Sigma-Aldrich, Poznań, Poland); 1-ethyl-3-(3-dimethylaminopropyl)carbodiimide (EDC), commercial grade, (Sigma-Aldrich, Poznań, Poland); acetonitrile, for HPLC-GC, ≥98.8% (GC) (Sigma-Aldrich, Poznań, Poland); PBS tablets, (Sigma-Aldrich, Poznań, Poland); glutaraldehyde (50 wt. % in H_2_O) (Sigma-Aldrich, Poznań, Poland); hexamethyldisilazane reagent grade (HMDS) (Sigma-Aldrich, Poznań, Poland); 96% ethyl alcohol (POCH, Gliwice, Poland); acetic acid (POCH, Gliwice, Poland); and potassium dihydrogen phosphate (POCH, Gliwice, Poland) were all used as received.

### 2.2. Etching of Halloysite Nanotubes

A total of 2.5 g of HNT was weighed, and then the sample was placed in a round bottom flask (500 mL), and 250 mL of 1 M acetic acid solution was added. The flask with the suspension was immersed in an oil bath placed on a magnetic stirrer. The reaction was carried out for 50 h at 50 °C with constant stirring. The mixture was then centrifuged (8500 rpm, 5 °C, 5 min), and the sediment was collected. The sediment (EHNT) was then resuspended in 40 mL of the distilled water using a vortex shaker and centrifuged again. The procedure of resuspending EHNT in fresh water, followed by the centrifugation, was continued until the pH of the collected supernatant was neutral. The obtained EHNT pellet was then frozen and lyophilized to obtain a dry solid.

### 2.3. Loading of CP into the HNT or EHNT

A total of 2 mL of CP (50 mg/mL) was added to 1 g of nanotubes (HNT or EHNT). The resulting suspension was thoroughly mixed using a vortex shaker. CP was loaded into the nanotubes under decreased pressure—the sample was placed in a vacuum desiccator at 25 °C, and the pressure was reduced in a controlled manner to 273 mbar. After 15 min, the pressure in the desiccator returned to ambient conditions. The sample was once again thoroughly mixed using a vortex shaker. The entire procedure was repeated two more times. Finally, the sample was centrifuged (8500 rpm, 5 °C, 5 min) and washed three times with distilled water. The sample was then frozen and lyophilized.

### 2.4. Hydrogel Preparation

#### 2.4.1. Preparation of Hydrogel H1-EDC/CaCl_2_

A total of 1 mL of 2% aqueous solution of sodium alginate and 1 mL of 4.88 mg/mL solution of collagen in acetic acid were mixed. Separately, 10 mg of β-TCP and 100 mg of EHNT-CP were mixed and suspended in 1 mL of distilled water and then added to the solution of the polymers. The resulting suspension was thoroughly mixed. A total of 100 µL of 6% CaCl_2_ was then added, and the suspension was mixed again, placed in the small mold and left for 10 min to settle. The obtained sample was frozen and freeze-dried. The dry sample was then placed in the 33 mM ethanolic solution of EDC and incubated for 24 h at 37 °C to allow for chemical cross-linking. Next, the sample was washed with water and placed in the 0.1 M aqueous solution of CaCl_2_ for 10 min to further physically cross-link the hydrogel. After this time, the resulting material, H1-EDC/CACl_2_, was removed from the solution, frozen and freeze-dried. 

#### 2.4.2. Preparation of Hydrogel H2-CACl_2_/EDC 

A total of 1 mL of 2% aqueous solution of sodium alginate and 1 mL of 4.88 mg/mL solution of collagen in acetic acid were mixed. Separately, 10 mg of β-TCP and 100 mg of EHNT-CP were mixed and suspended in 1 mL of distilled water and then added to the solution of the polymers. The resulting suspension was thoroughly mixed. A total of 100 µL of 6% CaCl_2_ was then added, and the suspension was mixed again, placed in the small mold and left for 10 min to settle. The obtained sample was frozen and freeze-dried. The dry sample was then placed in the 0.1 M aqueous solution of CaCl_2_ for 10 min. Next, the sample was washed with water, placed in the 33 mM ethanolic solution of EDC and incubated for 24 h at 37 °C. After this time, the resulting hydrogel H2-CaCl_2_/EDC was removed from the solution, frozen and freeze-dried.

### 2.5. Studies of the CP Release 

#### 2.5.1. Release from the Nanotubes (HNT-CP and EHNT-CP)

A total of 100 mg of the CP-loaded nanotubes (HNT-CP or EHNT-CP) were placed in the 15 mL falcon, and 2 mL of PBS were added. The sample was placed into the IKA KS 4000i control incubator and incubated at 37 °C with constant shaking. After determined periods of time, the sample was centrifuged at 9000 rpm for 5 min, and the supernatant was removed. Then, immediately, 2 mL of the fresh PBS were added to the solid residue, the sample was mixed using a Vortex shaker and the incubation was continued. The supernatants collected after each time were analyzed using HPLC to determine the amount of the released drug.

The loading efficiency and encapsulation efficiency values were calculated according to the equations presented below:

(1) $$\mathrm{LE}\;[\%]=\frac{\mathrm{weight}\;\mathrm{of}\;\mathrm{CP}\;\mathrm{in}\;\mathrm{the}\;\mathrm{sample}}{\mathrm{weight}\;\mathrm{of}\;\mathrm{the}\;\mathrm{sample}}\cdot 100\;\left[\%\right]$$

(2) $$\mathrm{EE}\;[\%]=\frac{\mathrm{weight}\;\mathrm{of}\;\mathrm{CP}\;\mathrm{entrapped}\;\mathrm{in}\;1\;\mathrm{mg}\;\mathrm{of}\;\mathrm{nanotubes}}{\mathrm{initial}\;\mathrm{weight}\;\mathrm{of}\;\mathrm{CP}\;\mathrm{added}\;\mathrm{to}\;1\;\mathrm{mg}\;\mathrm{of}\;\mathrm{nanotubes}}\cdot 100\;\left[\%\right]$$

#### 2.5.2. Release from H1-EDC/CaCl_2_ and H2-CACl2/EDC Hydrogels

A 50 mg sample of the ENTP-CP-loaded hydrogel (H1-EDC/CaCl_2_ or H2-CaCl_2_/EDC) was placed in the 15 mL falcon, and 2 mL of PBS were added. The sample was placed into the IKA KS 4000i control incubator and incubated at 37 °C with constant shaking. After the determined periods of time, the sample was centrifuged at 9000 rpm for 5 min, and the supernatant was removed. Then, immediately, 2 mL of the fresh PBS were added to the solid residue, the sample was mixed using a Vortex shaker and the incubation was continued. The supernatants collected after each time were filtered with the 45 µm PES syringe filter to remove any traces of polymers and analyzed using HPLC to determine the amount of the released drug. The drug was not retained by the syringe filter used, as confirmed experimentally.

### 2.6. Degradation Studies

The samples of H1-EDC/CaCl_2_ and H2-CaCl_2_/EDC were placed in the simulated body fluid (SBF) prepared according to Kokubo’s method [30] and incubated at 37 °C with constant shaking. After various time intervals, each sample was withdrawn from the SBF, the excess of the medium was removed, and the sample was weighted. The sample was then returned to the SBF, and the incubation was continued.

### 2.7. Biomineralization Studies

Biomineralization studies were performed in the SBF. The hydrogel (H1-EDC/CaCl_2_ and H2-CaCl_2_/EDC) samples were each placed in 5 mL of SBF. Then, the vials with the samples were sealed with parafilm and incubated at 37 °C for 7 or 14 days. Finally, the samples were washed with water and lyophilized.

### 2.8. SEM Measurements

The structure of the materials (CP-loaded nanotubes and EHNT-CP-containing hydrogels) and the morphology of the cells cultured on the hydrogels were visualized using a Hitachi S 4700 scanning electron microscope (FE-SEM). The freeze-dried samples were placed directly on the carbon tape attached to the holder and spatter coated with a thin layer of gold. 

### 2.9. TEM Measurements

Nanotubes (HNT-CP and EHNTS-CP) were also visualized using an FEI Tecnai Osiris (200 kV) transmission electron microscope (TEM). The average diameter of the nanotubes’ lumen for each sample was obtained from the analysis of the histograms generated based on the images collected in the STEM mode. The analysis was performed using DigitalMicrograph 3.0 software.

### 2.10. Antibacterial Tests

Discs with different amounts of CP or CP-containing nanotubes (HNT or EHNT) were prepared. For the studies on the hydrogels, the small discs of the hydrogels (H1-EDC/CaCl_2_ and H2-CaCl_2_/EDC), containing 5 mg of EHNT-CP each, were prepared according to the procedures described in Section 2.4, but with proportionally lower amounts of all components.

The *Staphylococcus aureus* strain NCTC8325 was grown overnight in Mueller Hinton Agar (MHA, Merck). It was then sieved onto the Mueller Hinton II broth and incubated at 37 °C until the log phase of growth was reached. The MHA plates were then inoculated with the broth of the strain. Blotted paper discs were arranged, and the test suspensions (CP, HNT-CP and EHNT-CP) were applied to the discs and incubated for 18 h. For the hydrogels, the prepared samples were placed on the blotted paper discs, wetted with 10 µL of water and incubated for 18 h.

### 2.11. MTT Test

Hs27 Human foreskin fibroblasts (ATCC), WM793 human melanoma (WM793—vertical growth phase—VGP) (ATCC) and human osteosarcoma (MG-63) (ATCC) cells were used in the studies. Hs27 is one of a series of human foreskin fibroblast lines developed at NBL, California; the material was obtained from a normal new-born, and the cells possess the G6PD type A phenotype. WM793 is a melanoma cell line from the vertical growth phase (VGP) of a primary skin melanoma lesion. It is representative of an early melanoma, with a low metastatic potential. MG-63, a cell line that has a fibroblast morphology, was isolated from the bone of a White, 14-year-old male patient with osteosarcoma.

For the biological tests, the two hydrogels (three samples for each type of hydrogel) were prepared in a 96-well plate, sterilized with UV radiation and incubated in the cell culture medium without serum for 1 h at 37 °C. Next, the medium was removed, and the Hs27, WM793 and MG-63 cells were seeded in the plate at a density of 5 × 10^4^ cells/well. The medium containing 90 vol% of DMEM (HS27 and MG-63 cells) or RPMI 1640 (WM793 cells) (supplemented with 1 vol% of penicillin-streptomycin solution) and 10 vol% of serum was used. The cells were cultured in the incubator (37 °C, 90% humidity with 5% CO_2_) according to a previously described procedure [31]. After 24 h and 48 h of culture, the cell viability was studied using the MTT assay according to the procedure used previously [32].

### 2.12. SEM Studies of the Cells’ Morphology on Hydrogels

Materials after 24 h of cell culturing were fixed and dehydrated. The hydrogels were washed with PBS once and next fixed using 2.5% glutaraldehyde in PBS for 75 min at room temperature. The glutaraldehyde was then discarded, and all samples were washed with PBS. In the next step, samples were dehydrated using 200 µL of 60%, 70%, 80% and 90% of ethanol (for 5 min each) and with absolute ethanol for 7 min. Then, the samples were immersed and kept in HMDS for 15 min and finally air-dried at room temperature [4,33].

### 2.13. Other Measurements

The pore size distribution of the lyophilized H1-EDC/CaCl_2_ and H2-CaCl_2_/EDC hydrogels was measured using a mercury porosimeter (PoreMaster 60, Quantachrome Inc., Warsaw, Poland). The Nicolet iS10 FTIR spectrometer (Thermo Fischer, Warsaw, Poland) with an attenuated total reflection accessory (Attenuated Total Reflectance, ATR; SMART iTX) (Thermo Fischer, Warsaw, Poland) was used to evaluate the results of the nanotubes’ etching process and to study the hydrogels after biomineralization. The thermogravimetric analysis of the HNT-CP and EHNT-CP samples was performed on an 851e TGA/SDTA microthermogravimeter equipped with QMS Thermostar GSD 300 T Balzers (Mettler Toledo, Warsaw, Poland) in the temperature range of 25 to 600 °C, with a heating rate of 10 °C/min, under nitrogen atmosphere.

## 3. Results and Discussion

### 3.1. Bioactive EHNT-CP System

#### 3.1.1. Pre-Treatment and Drug Loading of Halloysite Nanotubes

Halloysite nanotubes allow for an effective encapsulation of negatively charged drugs in their positively charged interior by taking advantage of the electrostatic interactions. The amount of the drug loaded is, however, limited by the size of the lumen. In order to increase the loading efficiency of the HNT interior, it was additionally etched with acetic acid based on the procedure described in the previous literature report by Garcia-Garcia [34]. Clindamycin was then loaded into the modified halloysite nanotubes (EHNT) under reduced pressure using a previously developed methodology [35].

**Figure 1:**
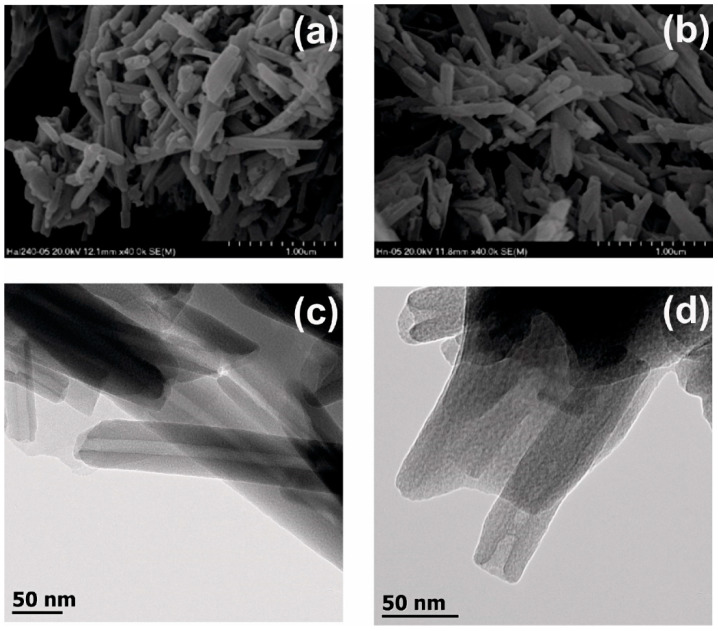
(**a**) SEM image obtained for HNT-CP, (**b**) SEM image obtained for EHNT-CP, (**c**) TEM image obtained for HNT-CP and (**d**) TEM image obtained for EHNT-CP.

In order to verify the possible negative influence of the etching process, the morphology of HNT and EHNT was examined using SEM and TEM; the latter allowed us to also evaluate the change in the size of the nanotube’s lumen. The SEM images (Figure 1a,b) of both materials did not show any significant differences—both HNT and EHNT had a smooth outer surface, an elongated shape and sizes in the range of 200 nm–1200 nm. The TEM analysis (Figure 1c,d) confirmed that both the outer and inner surfaces of the nanotubes remained intact, while the average size of the lumen was increased from 16.1 nm to 17.8 nm. The ATR-FTIR spectra of HNT and EHNT showed no differences between acid-treated and raw halloysite (Supplementary Materials, Figure S1).

**Figure 2:**
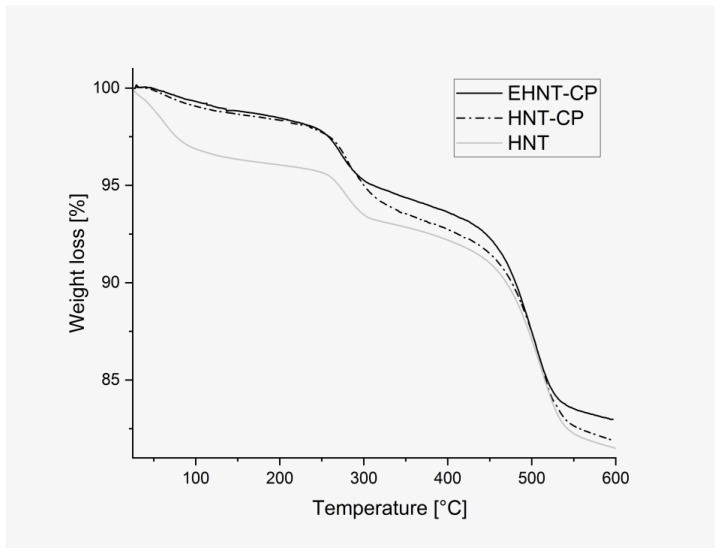
Thermogravimetric curves for HNT, HNT-CP and EHNT-CP.

To study the changes in the HNT due to the etching process and the further introduction of CP, the results of the thermogravimetric analysis were compared for all the above materials (Figure 2). For raw HNT, the first decrease in the weight was observed in the temperature range of 30 °C–100 °C and can be ascribed to the loss of adsorbed water. This change is not visible for HNT-CP and EHNT-CP materials, as they were both lyophilized after CP loading and stored in moist-free conditions. The slow, monotonous decrease in weight is visible for both CP-containing materials in the temperature range of 40 °C–250 °C, which is due to the decomposition of the nanotube-protected antibiotic. According to the literature data [36], the thermal decomposition of the clindamycin phosphate introduced into the halloysite nanotubes was observed in a similar temperature range. At first, CP was degrading slightly faster in the case of unmodified nanotubes, but the degradation was over at ca 250 °C. For the EHNT system, it continued up to 280 °C, and at the end of this process, the line for EHNT-CP dropped below that of HNT-CP material, suggesting that more of the antibiotic was entrapped in the former. Importantly, as the unprotected clindamycin decomposes at ca 220 °C [37], the stabilizing effect of the nanotubes is confirmed for both (HNT-CP and EHNT-CP) systems. Between 250 °C and 450 °C, another slope is observed, which indicates the loss of the water bound inside the structure of the halloysite. Here, we observe that less water was bound inside EHNT-CP, most probably due to the fact that the walls of EHNT nanotubes consist of fewer layers after the etching process. The weight loss registered in the range of 450 °C–600 °C can be attributed to the removal of the halloysite hydroxyl groups [38] and was similar for all materials. 

#### 3.1.2. Studies of CP Release from HNT and EHNT Nanotubes 

**Figure 3:**
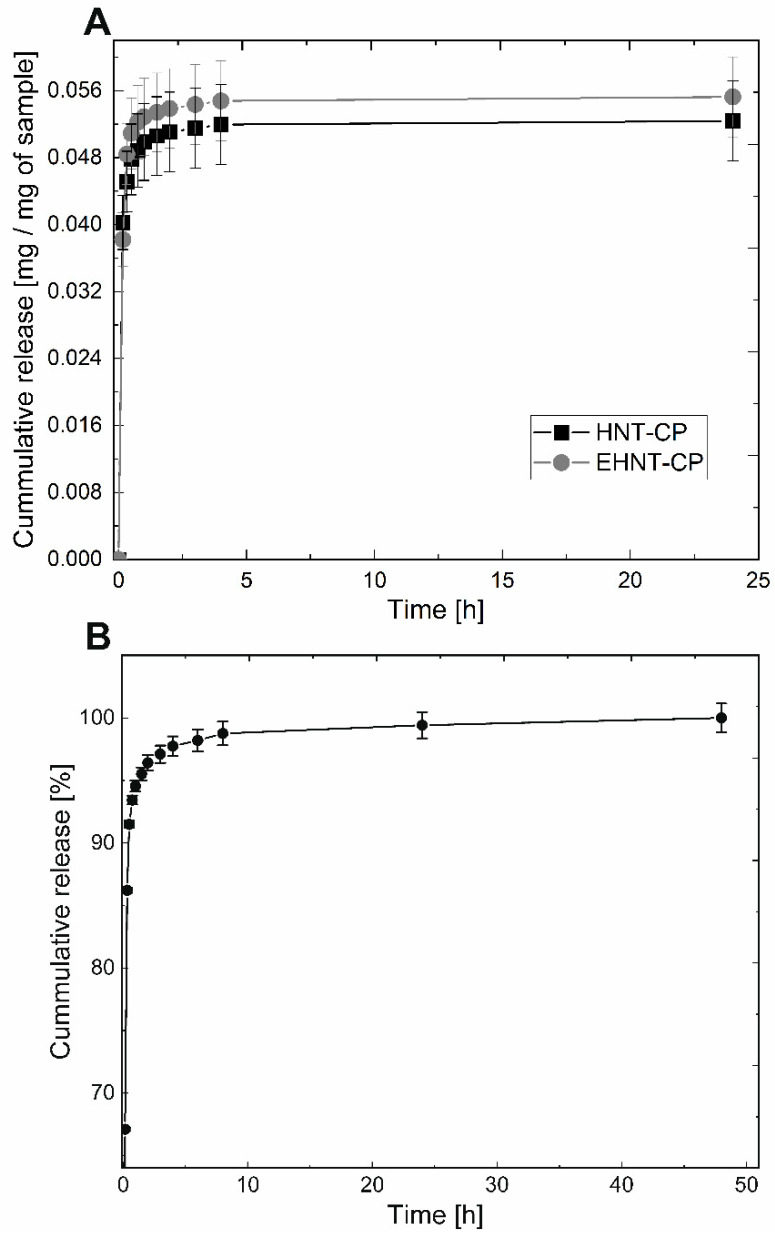
(**A**) Release profiles for HNT-CP and EHNT-CP; (**B**) extended-release profile for EHNT-CP.

The CP release profiles for both the HNT-CP and EHNT-CP systems are presented in Figure 3A. A significant “burst” release was observed for both systems, most probably due to the presence of the drug physically adsorbed on the outer surface of the nanotubes. The observed release was fast during the first 45 min, slightly slower for another 3 h and then even slower; CP was, however, still being released from both types of the nanotubes for up to 24 h. After 24 h, as expected, the amount of the drug released from the 1 mg of the modified nanotubes was higher (0.055 mg vs. 0.052 mg for unmodified HNT). For the EHNT-CP system, the drug continued to be released for another 24 h, while in the case of HNT-CP, no more release was observed after this time. The extended release profile obtained for the EHNT-CP system is presented in Figure 3B. Based on the amount of CP released, the loading efficiency (LE%) and encapsulation efficiency (EE%) values were calculated for both systems: 5.2% and 34.2% for HNT-CP, and 6.1% and 40.2% for EHNT-CP, respectively. The obtained release profiles allow for the rapid initial increase in the CP concentration in the local environment, followed by the prolonged release of the small amounts of the drug to sustain its local concentration level for a longer time period.

#### 3.1.3. Antibacterial Properties of HNT-CP and EHNT-CP

**Figure 4:**
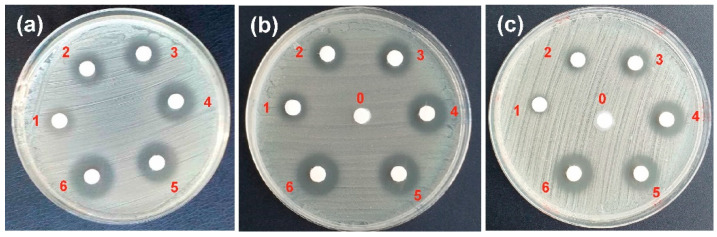
Growth inhibition zones obtained for the Staphylococcus aureus strain grown on the Mueller Hinton II medium after the application of various amounts of: (**a**) CP, (**b**) HNT-CP, (**c**) EHNT-CP (see Table 1).

The antibacterial activity of halloysite nanotubes with immobilized clindamycin phosphate was investigated using the Staphylococcus aureus strain grown on the Mueller Hinton II medium. Paper discs with different amounts of free CP, HNT-CP and EHNT-CP were placed on the plates with the cultured bacterial strains, and the increasing diameters of the bacteria growth inhibition zones were observed for each of the systems after 18 h of incubation (Figure 4). To verify if the carrier itself (drug-free nanotubes) exhibits the antibacterial properties, which could interfere with the measurement, “empty” HNT/EHNT were also tested (see the samples marked with “0” in Figure 4b,c). The weights of the samples and the calculated CP content in each sample of HNT-CP and EHNT-CP are presented in Table 1.

The absence of the growth inhibition zones around “empty” HNT/EHNT samples confirmed that the “empty” nanotubes did not exhibit any bactericidal properties. The comparison between the inhibition zones observed for the same amounts of HNT-CP and EHNT-CP revealed no significant differences. The inhibition zones for the free CP samples containing twice as much of the drug as the samples of HNT-CP/EHNT-CP (Table 1) were similar. It may thus be concluded that the nanotubes had no negative effect on the biological activity of the antibiotic. On the contrary, the slower release of CP from the nanotubes improved the overall efficacy of CP against *Staphylococcus aureus* bacteria.

### 3.2. Bioactive Alginate-Based Hydrogels 

The analysis of the results obtained for the HNT-CP and EHNT-CP systems showed that the latter had higher LE and EE values and released the antibiotic for a longer time. At the same time, both HNT and EHNT provided CP with increased thermal stability and positively influenced its antibacterial properties. The EHNT-CP system was therefore chosen to be tested as an antibacterial component of the scaffold. 

Based on the previous reports [39] and the preliminary experiments, the weight ratio of sodium alginate, collagen I and β-TCP was set as 4:1:2. Halloysite was then added to the composition in the five different EHNT-CP/alginate weight ratios (2.5, 4.5, 5.0, 5.5), and the obtained sol was physically crosslinked with calcium ions. The highest amount of EHNT-CP that did not negatively influence hydrogel’s homogeneity and integrity was determined, and based on the result, the sodium-alginate-to-EHNT-CP-weight ratio was set to 0.3. In order to improve the stability of the hydrogel, we have decided to complement the physical crosslinking between the sodium alginate chains with the chemical crosslinking via carbodiimide chemistry, using two different approaches. In the first one, the pre-settled and freeze-dried hydrogel was first incubated for 24 h in the ethanolic solution of EDC to allow for the chemical crosslinking between the carboxylic groups of the alginate and the amine groups of the collagen. As the reaction needs time, in order to avoid the unnecessary release of CP, an ethanolic solution of EDC was used [40]. In the next step, the sample was washed with water and placed in the aqueous solution of CaCl_2_ for 10 min to allow for the physical crosslinking of alginate chains, resulting in the hydrogel H1-EDC/CaCl_2_. As an alternative, to obtain hydrogel H2-CaCl_2_/EDC, the scaffold was crosslinked—first physically in an aqueous solution of CaCl_2_ and then chemically in an ethanolic solution of EDC.

#### 3.2.1. Morphology and Porosity of the Hydrogels

**Figure 5:**
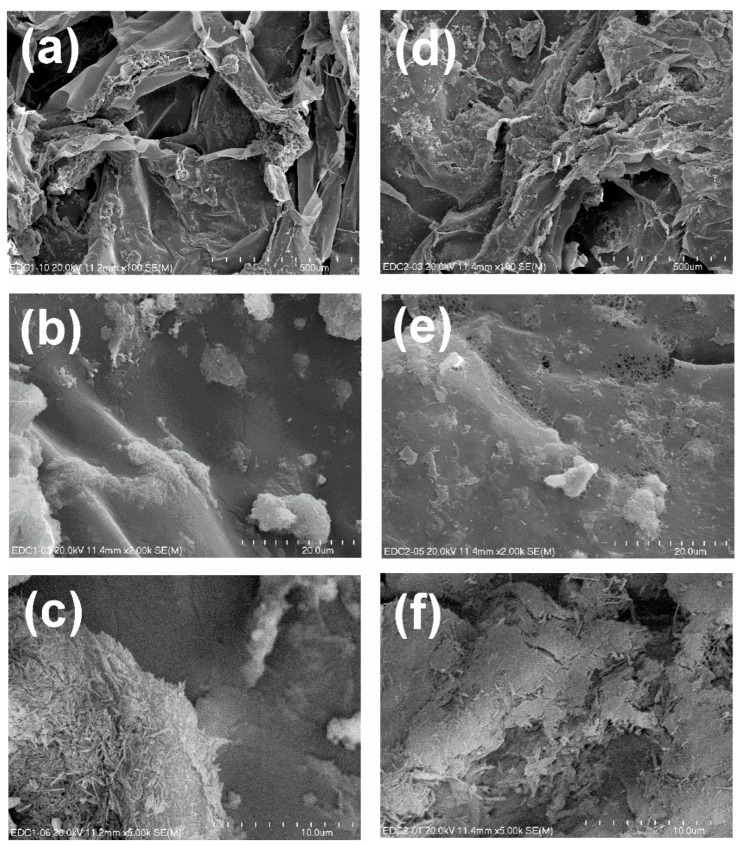
(**a**–**c**) SEM images obtained for the hydrogel H1-EDC/CaCl_2_; (**d**–**f**) SEM images obtained for the hydrogel H2-CaCl_2_ /EDC.

The morphology of both hydrogels, H1-EDC/CaCl_2_ and H2-CaCl_2_/EDC, was studied based on the SEM images shown in Figure 5. The obtained hydrogels showed a porous structure, with the pores in the range of 50 µm–500 µm (Figure 5a,d). The H1-EDC/CaCl_2_ morphology suggested that it was slightly more porous then H2-CaCl_2_/EDC. The images presented in Figure 5b,e revealed a much more homogenic distribution of EHNT-CP in the H2-CaCl_2_/EDC hydrogel in comparison with H1-EDC/CaCl_2_. The same can be observed when images c and f are compared. 

The porosity studies have confirmed the observations based on the SEM analysis. Most of the pores were in the size range of 20 µm–200 µm. Both materials were characterized by a high porosity, which was found to be 92.41% for H1-EDC/CaCl_2_ and 87.25% for H2-CaCl_2_/EDC. Thus, the former hydrogel was slightly more porous. This may be correlated with the distribution of the nanotubes within the hydrogel matrix. In the H2-CaCl_2_/EDC material, EHNT-CP is distributed more evenly, and fewer large agglomerates were observed. It is thus possible that some of the nanotubes filled in the smallest pores, reducing the porosity of the hydrogel. The pore size distributions obtained for both materials are presented in the Supplementary Materials (Figure S2).

#### 3.2.2. Drug Release from Hydrogels

**Figure 6:**
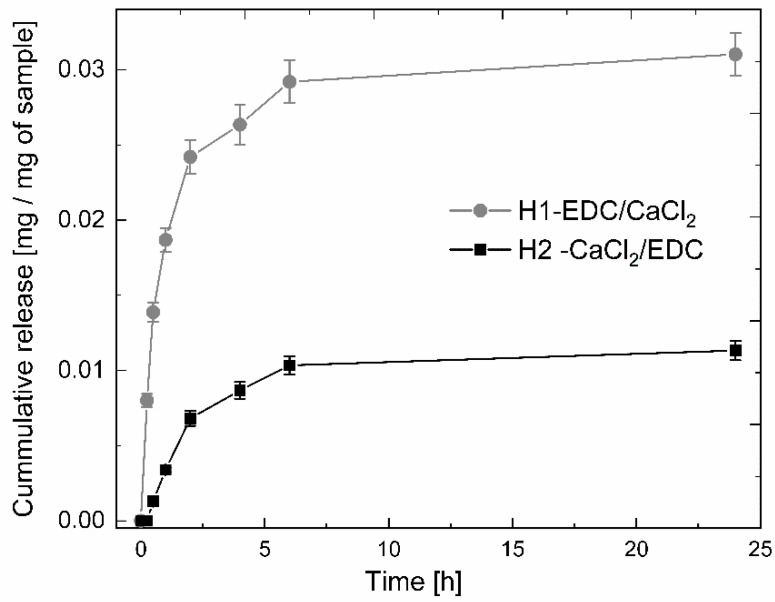
Release profiles obtained for the hydrogels H1-EDC/CaCl_2_ and H2-CaCl_2_/EDC.

The comparison of the release profiles for H1-EDC/CaCl_2_ and H2- CaCl_2_/EDC in PBS (pH = 7.4) and at physiological temperatures revealed that the shapes of both are similar with the fast, linear release for the first 2 h, slower for another 5 h and slow but still visible after 24 h (see Figure 6). The amount of CP released, however, differed significantly—within the first 24 h, 1 mg of hydrogel H1-EDC/CaCl_2_ released ca 30 μg of CP, while only ca 10 μg of the antibiotic was released from H2-CaCl_2_/EDC in the same time. This result confirmed our concern about the significant loss of the drug during the crosslinking process in aqueous conditions. As the initial crosslinking of H2-CaCl_2_/EDC was conducted in aqueous conditions, more of the drug was effectively lost during this process, while in the case of H1-EDC/CaCl_2_, the initial chemical crosslinking in the ethanolic solution prevented the significant loss of CP during the 15 min incubation in the aqueous solution of calcium chloride. Additionally, the more homogenous distribution of the nanotubes in the polymeric matrix of H2-CaCl_2_/EDC may result in the more effective coating of each nanotube by the polymers, resulting in the overall slower release rate. The latter phenomenon is further supported by the less steep slope of the release profile observed for hydrogel H2-CaCl_2_/EDC during the first 2 h. 

#### 3.2.3. Swelling and Degradation Studies

The swelling degree for both of the studied hydrogels was estimated in SBF to mimic the physiological conditions. The obtained swelling ratios were well above 100%, confirming the hydrogel character of the obtained material. The swelling ratio was higher for H1-EDC/CaCl_2_ (798%) then it was for H1-CaCl_2_/EDC (653%), which was consistent with the higher porosity found for the former material. The more porous structure of the hydrogel allowed for a better penetration by SBF, leading to the higher swelling. 

**Figure 7:**
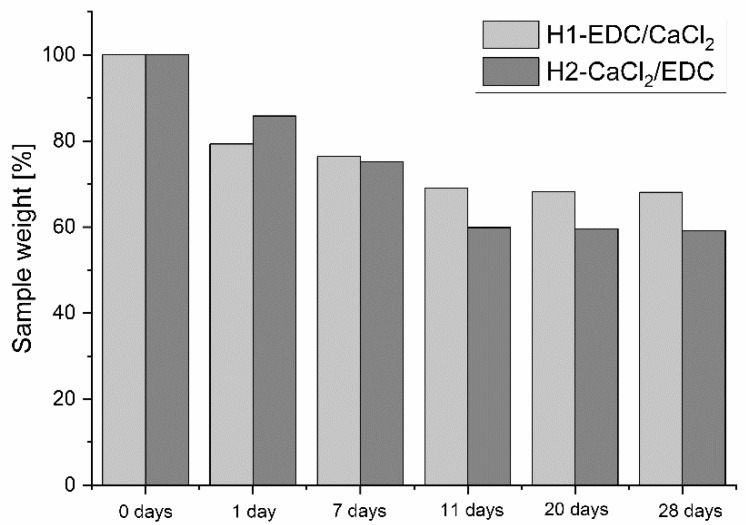
Results of the degradation studies for H1-DEC/CaCl_2_ and H2-CCl_2_/EDC in SBF at 37 °C.

For the material in contact with physiological fluids, it is important to study its degradation process. The results of the degradation studies are presented in Figure 7. While for the first 24 h, the hydrogel H1-EDC/CaCl_2_ degraded somewhat faster, most probably due to the higher porosity and the more effective swelling, the results obtained after 7, 11, 20 and 28 days showed a faster degradation of H2-CaCl_2_/EDC. Both hydrogels degraded slowly and only partially within the 28 days’ time period. The weight loss after 4 weeks was below 41% for both hydrogels, and the changes observed after day 11 were small. 

#### 3.2.4. Antibacterial Activity of the Hydrogels

**Figure 8:**
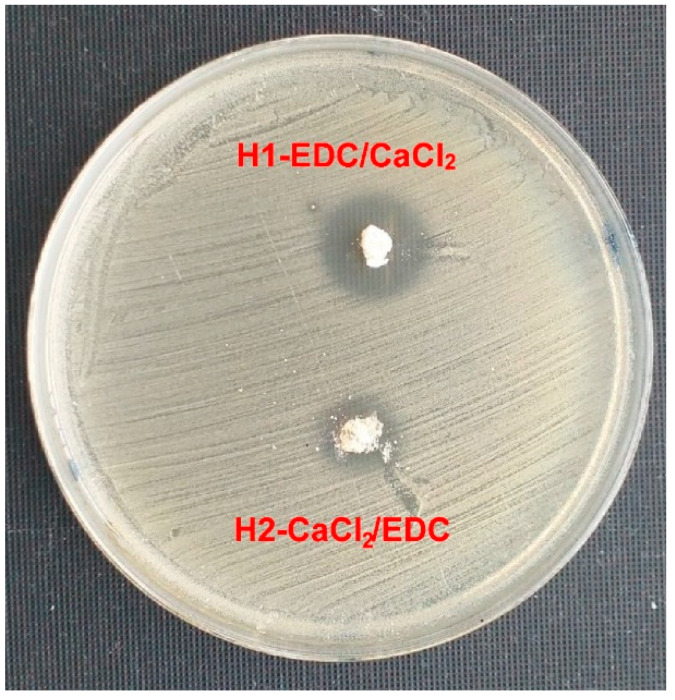
Growth inhibition zones obtained for the *Staphylococcus aureus* strain grown on the Mueller Hinton II medium after the application of H1-EDC/CaCl_2_ and H2-CaCl_2_/EDC hydrogels.

The antibacterial activity of both hydrogels was also evaluated. Discs of both materials containing 5 mg of the EHNT-CP were studied, similar to the EHNT-CP nanotubes (Figure 8). Based on the estimated amount of CP entrapped in 1 mg of EHNT (0.0553 mg), each disc should contain ca 0.28 mg of CP. The diameters of the bacteria growth inhibition zones observed for both materials differed significantly. While the zone for H1-EDC/CaCl_2_ was similar to the one obtained for 0.1 mg of free CP, the diameter of the zone observed for H2-CaCl_2_/EDC was much smaller, close to the result obtained for 0.025 mg of free CP. These results were in agreement with the amounts of CP released from both materials and suggested that the procedure of preparing the H2-CaCl_2_/EDC hydrogel led to a bigger loss of the drug. Still, both hydrogels show antibacterial activity. 

#### 3.2.5. Biomineralization Studies

**Figure 9:**
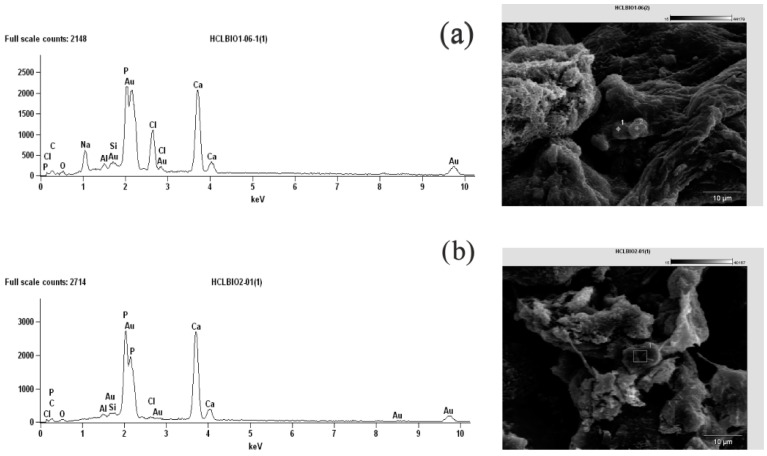
EDX analysis (left panel) and SEM images (right panel) obtained after 7 days of the biomineralization process for: (**a**) the hydrogel H1-EDC/CaCl_2_; (**b**) the hydrogel H2-CaCl_2_/EDC.

One of the important properties of the materials designed for bone repair is their ability to promote the biomineralization process. As the proposed hydrogels contained collagen type I and β-TCP, which are both known to promote biomineralization, we have verified the ability of our hydrogels to induce the formation of the biomineral. The samples of both materials were incubated for 7 and 14 days in SBF solution at a physiological temperature (37 °C). The SEM analysis has confirmed that, in both samples, the characteristic, cauliflower-like structures of calcium phosphate could be found (Figure 9). As can be seen in the SEM pictures, the structure and the stability of the hydrogels were preserved. 

The EDX analysis after 7 days of biomineralization resulted in a Ca/P ratio of 1.72 (Ca wt% = 21.34 and P wt% = 12.44) for H1-EDC/CaCl2 and a Ca/P ratio of 1.79 (Ca wt% = 32.76 and P wt% = 18.28) for H2-CaCl2/EDC. While the Ca/P value for the pure hydroxyapatite phase is 1.67, the mineral phase found in the bones is often in excess of this value due to the impurities of the calcium carbonate [41,42]. The Ca/P value of 1.75 is often reported for natural hydroxyapatite, and the values obtained for the crystals found in our hydrogels are both close to this value, suggesting that at least small amounts of the hydroxyapatite were indeed formed. 

**Figure 10:**
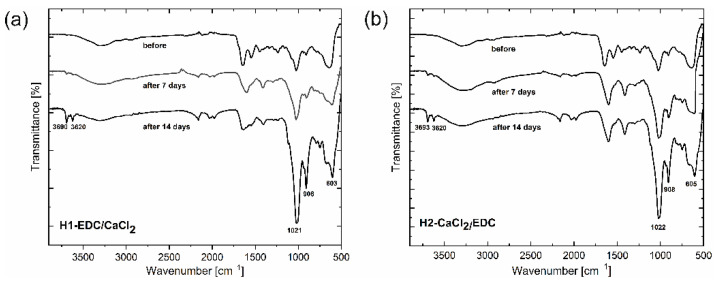
FTIR spectra of (**a**) the hydrogel H1-EDC/CaCl_2_ and (**b**) the hydrogel H2-CaCl_2_/EDC before and after 7 and 14 days of incubation in SBF at 37 °C.

The ATR-FTIR spectra of both hydrogels before biomineralization and after 7 and 14 days of incubation in SBF are shown in Figure 10. 

For both hydrogels, there is a significant change in the intensity of the bands at 1021–1022 cm^−1^ and at 603–610 cm^−1^ after 7 and 14 days of incubation in SBF, compared to the same material before biomineralization. As these bands can be assigned to the vibrations of PO_4_^3−^ ions [43], the observed increase confirms the formation of the calcium phosphate in the biomineralization process. There is also a much less pronounced increase observed in the intensity of the bands at 908 cm^−1^, 3620 cm^−1^ and 3690 cm^−1^, which can be correlated with the inner surface -OH groups bending and stretching vibrations of HNT [44]. This small change may be explained by the slow degradation of the polymeric matrix, leading to the relative increase in the intensity of the inorganic part of the hydrogel. The increase is, however, too small to account for the observed changes in the bands’ intensities in the aforementioned region, which are characteristic for the PO_4_^3−^ ions.

#### 3.2.6. Biological Studies of the Bioactive Hydrogels

**Figure 11:**
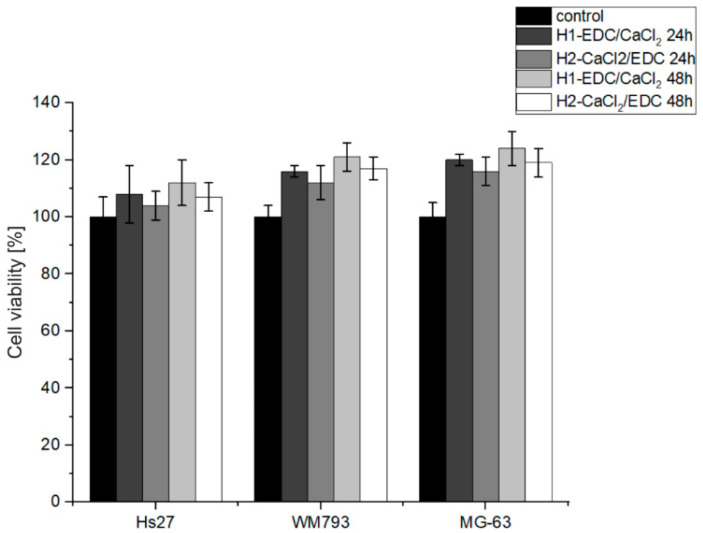
The results of the MTT assay performed for both hydrogels on the Hs27, WM793 and MG-63 cell lines.

The cytotoxicity of the H1-EDC/CaCl_2_ and H2-CaCl_2_/EDC hydrogels was established toward MG-63 human osteosarcoma, Hs-27 human foreskin fibroblasts and WM-793 human melanoma cell lines using the MTT cell viability assay. The test was carried out on both cell lines for 24 and 48 h. The obtained results (see Figure 11) did not indicate any cytotoxic effect on the tested cells. For both hydrogels, the cell viability after 24 h and 48 h was above 100% (control) for all the tested cell lines. This may be explained by the presence of collagen, which is a natural component of the bone. Further, slightly better results were observed for the H1-EDC/CaCl_2_ hydrogel. This may be explained by the fact that the traces of potentially cytotoxic ethanol could be additionally washed out during incubation in the aqueous solution of calcium chloride. The most promising results were obtained for the MG-63 osteoblast-like cell line, which is often used as a model for osteoblasts. Both materials also promoted cell proliferation, which was quite visible after 48 h. 

**Figure 12:**
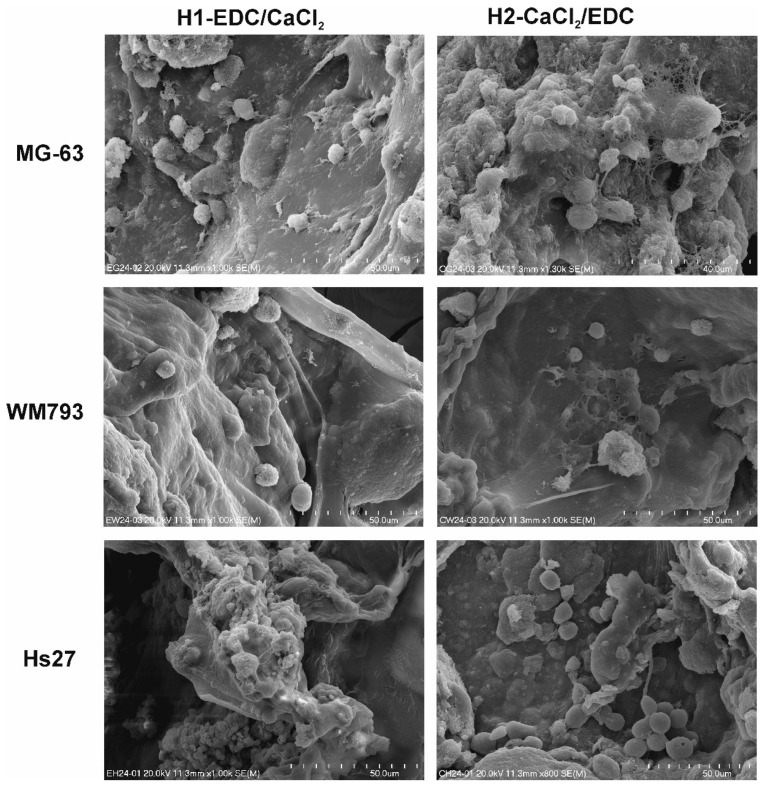
SEM images of the MG-63, HS-27 and WM-973 cell lines cultured on the H1-EDC/CaCl_2_ and H2-CaCl_2_/EDC hydrogels for 24 h.

To give an insight into the morphology and condition of the cells seeded on our hydrogels and incubated in the conditions identical to those of the MTT assay, the SEM images after 24 h of incubation are presented in Figure 12. Both types of hydrogels are well populated by the cells of all the tested lines. All three cell types adhere to the surface, and some of the cells, especially MG-63, started to spread and flatten on the surface of the material.

## 4. Conclusions

CP was effectively entrapped in the lumen of both raw (HNT) and etched (EHNT) halloysite nanotubes. The etching process did not influence the morphology of the HNT but allowed for the enlargement of their lumen, as observed by SEM and TEM studies. CP was released from both HNT and EHNT in a controlled manner. Based on the release profiles, the loading efficiencies for both types of nanotubes were determined as 5.2% and 6.1% for HNT and EHNT, respectively. These results confirmed the positive effect of the etching process on the amount of the drug entrapped in the nanotubes. As more of the antibiotic was entrapped in EHNT, and CP was released for a longer time from these nanotubes, we chose the EHNT-CP system for further studies. 

To test EHNT-CP as an antibacterial component of the material for bone regeneration, two hydrogels were prepared. Both materials had the same composition, which consisted of sodium alginate, collagen, β-TCP and EHNT-CP, but they were crosslinked using two different procedures. EHNT-CP was successfully incorporated into both hydrogels. Both hydrogels were highly porous and effectively promoted biomineralization. The differences introduced by a crosslinking process had, however, an impact on the porosity of the materials, the distribution of EHNT-CP nanotubes within the hydrogel matrix and, consequently, the CP release profile. While the EHNT-CP system was distributed more evenly in the hydrogel H2-CaCl_2_/EDC, the hydrogel H1-EDC/CaCl_2_ showed a higher porosity and a more advantageous release. The latter also degraded slower in the SBF, and after 28 days, the loss of its weight was well below 40%. Antibacterial studies confirmed the significant activity of both the HNT-CP and EHNT-CP systems, as well as both hydrogels, with the hydrogel H1-EDC/CaCl_2_ showing significantly better antibacterial properties. The cytocompatibility of both materials was also confirmed. Thus, the potential of both hydrogels in bone repair applications was shown, with the H1-EDC/CaCl_2_ hydrogel exhibiting more favorable properties. At the same time, the possibility of using the EHNT-CP system as a bioactive component of such material was positively verified. 

## Figures and Tables

**Table 1:** The weights of the samples of free CP, HNT-CP and EHNT-CP used in the antibacterial test (see Figure 4) and the calculated content of CP in HNT-CP and EHNT-CP samples.

Sample Number	Weight of CP (Solution) [mg]	HNT-CP		EHNT-CP	
		Sample Weight [mg]	CP Weight in the Sample [mg]	Sample Weight [mg]	CP Weight in the Sample [mg]
1	0.0250	0.2500	0.0131	0.2500	0.0138
2	0.0375	0.3750	0.0197	0.3750	0.0207
3	0.0500	0.5000	0.0262	0.5000	0.0276
4	0.0625	0.6250	0.0327	0.6250	0.0345
5	0.0750	0.7500	0.0393	0.7500	0.0414
6	0.1000	1.0000	0.0524	1.0000	0.0553

## Data Availability

The data presented in this study are available on request from the corresponding author.

## Supplementary Materials

The following supporting information can be downloaded at: https://www.mdpi.com/article/10.3390/polym14235151/s1, Figure S1: FTIR spectra of HNT and EHNT; Figure S2: Porosimetry histograms for the hydrogels H1-EDC/CaCl_2_ and H2-CaCl_2_/EDC.
