# Supplementary material for: Clindamycin-Loaded Halloysite Nanotubes as the Antibacterial Component of Composite Hydrogel for Bone Repair
Source: Polymers (Basel). 2022 Nov 26;14(23):5151. doi: 10.3390/polym14235151 (PMC9739121; doi:10.3390/polym14235151)
Supplement: Supplementary file 1 [file polymers-14-05151-s001.zip › polymers-1955805-supplementary.pdf]

Supplementary Materials

“Clindamycin-loaded, acid-etched halloysite nanotubes as the antibacterial component of the multicomponent hydrogel for bone repair”

Adrianna Machowska, Joanna Klara, Gabriela Ledwójcik, Kinga Wójcik, Joanna Dulińska-Litewka, Anna Karewicz

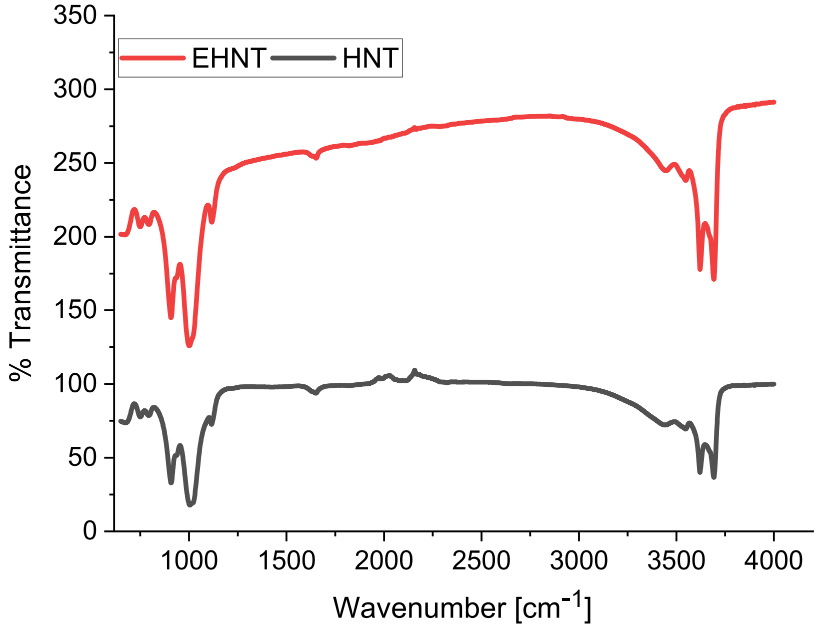

Figure S1. FTIR spectra of HNT and EHNT

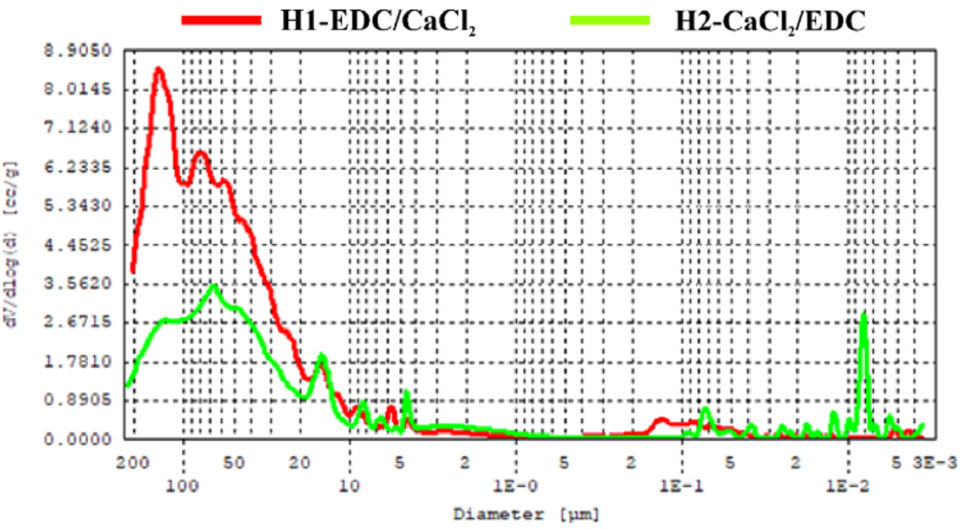

Figure S2. Porosimetry histograms for hydrogels H1-EDC/$CaCl_2$ and H2-$CaCl_2$/EDC
